# Reliability Prediction of Acrylonitrile O-Ring for Nuclear Power Applications Based on Shore Hardness Measurements

**DOI:** 10.3390/polym13060943

**Published:** 2021-03-19

**Authors:** Alvaro Rodríguez-Prieto, Ernesto Primera, Mariaenrica Frigione, Ana María Camacho

**Affiliations:** 1 Department of Manufacturing Engineering, Universidad Nacional de Educación a Distancia (UNED), 28040 Madrid, Spain; amcamacho@ind.uned.es; 2 Department of Industrial Inspection and Technical Assistance, SGS Tecnos, 28042 Madrid, Spain; 3 Department of Applied Statistics, University of Delaware, 531 South College Avenue, Newark, DE 19716, USA; eprimera@udel.edu; 4 Machinery and Reliability Institute (MRI), 2149 Adair Ct. Mobile, AL 36695, USA; 5 Department of Engineering for Innovation, University of Salento, Prov. le Lecce-Monteroni, 73100 Lecce, Italy; mariaenrica.frigione@unisalento.it

**Keywords:** reliability, prognostics, design-for-reliability, aging, elastomers, durability, harsh environments

## Abstract

The degradation of polymeric components is of considerable interest to the nuclear industry and its regulatory bodies. The objective of this work was the development of a methodology to determine the useful life—based on the storage temperature—of acrylonitrile O-rings used as mechanical sealing elements to prevent leakages in nuclear equipment. To this aim, a reliability-based approach that allows prediction of the use-suitability of different storage scenarios (that involve different storage times and temperatures) considering the further required in-service performance, is presented. Thus, experimental measurements of Shore A hardness have been correlated with storage variables (temperature and storage time). The storage (and its associated hardening) was proved to have a direct effect on in-service durability, reducing this by up to 60.40%. Based on this model, the in-service performance was predicted; after the first three years of operation the increase in probability of failure (POF) was practically insignificant. Nevertheless, from this point on, and especially, from 5 years of operation, the POF increased from 10% to 20% at approximately 6 years (for new and stored). From the study, it was verified that for any of the analysis scenarios, the limit established criterion was above that of the storage time premise considered in usual nuclear industry practices. The novelty of this work is that from a non-destructive test, like a Shore A hardness measurement, the useful life and reliability of O-rings can be estimated and be, accordingly, a decision tool that allows for improvement in the management of maintenance of safety-related equipment. Finally, it was proved that the storage strategies of our nuclear power plants are successful, perfectly meeting the expectations of suitability and functionality of the components when they are installed after storage.

## 1. Introduction

The mechanical characterization of materials provides the basis for the fundamental understanding of the behavior of components that can experience degradation in operation and/or during storage. A representative example is the thermal aging mechanism that severely affects materials that are ultimately intended to operate in the harsh service environment of a nuclear reactor. Materials based on organic polymers have many applications (sealings, insulations, etc.) in nuclear power plants (NPPs).

Nowadays, polymer materials hold an important role in the industry, thanks to their unique properties, such as a wide range of operating temperatures, high thermal/electrical insulation, corrosion- and light-resistance, and sufficient mechanical properties (high strength-to-weight ratio, stiffness, toughness, and ductility) [1,2].

In addition, in some applications, the functionality of a polymeric component can be crucial for the safe operation of the plant [3]. The degradation of such components is therefore of considerable interest to the nuclear industry and its regulatory bodies, generating a large number of studies worldwide [4]. Elastomers are widely used in industry and in particular are often applied in sealing due to their ability to undergo high elastic deformation [5]. Synthetic and natural polymers normally degrade during their service-life, due to the exposure to different environmental conditions [6]. The degradation of polymeric materials is a frequent phenomenon that is accelerated, in many cases, by arduous operating conditions. Being able to predict the lifetime of elastomers is fundamental for many industrial applications [7].

Prognostics and health monitoring (PHM) analysis requires several stages, including data collection, data processing, condition monitoring, diagnostics, prognostics, and decision support [8]. The information generated by a PHM system can be divided into diagnostics and prognostics—diagnostics include anomaly detection, fault isolation, and fault classification and its uncertainty [9], while prognostics include the estimation of the remaining useful life (RUL) and the prediction of behavior at design stage. These procedures allows us to be sure that the component is in a good condition before installation and operation [10]. One focus of ongoing research is the identification of new indicators of polymer aging, which may be measured nondestructively, and used to predict of further behavior [11]. One of these is the non-destructive procedure to determine the Shore hardness. The mechanical properties are critically important for demanding applications. These include materials hardness since this property is strongly dependent on the operation (or even storage) parameters, the composition of the material, and the manufacturing process [12].

One of most usual parts with relevant safety-related function in nuclear equipment is the acrylonitrile (NBR) O-rings that are used as mechanical sealing elements since their safety function is capable of preventing any leakage (whether internal or external) throughout the useful life of the equipment [13]. NBR exhibits a relatively low density, moderate tensile strength, and high oil resistance [14,15,16]. O-rings are really the most common type of sealing used in industry due to their robustness, versatility, and low cost. The end-users typically receive only the end part which needs to be tested [17]. In nuclear plants, Shore A hardness tests are usually performed when O-rings are received and/or when they are installed.

Reliability evaluation plays an important role in the design and development of any engineering system [18]; thus, some studies [19,20] have correlated the main polymer properties with final performance and durability. Lifetime prediction of elastomer components is a very challenging task due to different factors. Determining a suitable and reliable end-of-lifetime criterion for O-ring seals is an important issue for long-term seal applications [21]. Ageing is a term used in many branches of polymer science and engineering when the properties of the polymer change over a period of time [22,23].

Polymers, and especially elastomers, play a key role as part of the many mechanical, electrical, and electronic components found in nuclear power generation plants [24]. Condition monitoring and an understanding of the degradation processes due to short-term thermal stress have been of interest to the nuclear industry because of qualification requirements [25]. Elastomers, especially rubbers—such as acrylonitrile butadiene (NBR)—experience degradation that is favored by contact with oxygen [26]. This type of reaction—which triggers the irreversible damage of the component—is also favored by an increase in the operating temperature. Therefore, it is of interest to analyze how the intrinsic properties of elastomers influence their thermal aging. When elastomers are exposed to environmental conditions, their functionality in operation might be limited due to degradation [27]. The accurate prediction of the mechanical properties of polymers is important for preventing industrial accidents while operating a machine. In general reactions, the linear Arrhenius equation is used to predict the aging characteristics [28].

The objective of this work is the development of a methodology to determine the useful life—based on the storage temperature—of NBR O-rings using a reliability-based approach that allows us to obtain the health condition at different supposed storage scenarios, considering the required in-service performance. For this study, NBR was selected as a gasket material, since a previous work [20] has shown that acrylonitrile is the best option to withstand moderate levels of radiation thresholds extracted from databases [29,30] as well as its recyclability, providing a sustainable life cycle. The evaluated parameter was the Shore A hardness in accordance with ISO 868 [31] during a period of five years. Measurements of Shore A hardness consisted of vertical immersion of the indenter into the composite surface [32]. The thermal hardening was quantified based on an adaptation of Arrhenius model-based correlation between hardness and temperature and storage time. This study incorporates a comparison between the results obtained for recently manufactured and existing O-rings in the warehouse, considering several statistical scenarios.

Using an adaptation of the Arrhenius model, predictions based on hardness results can be made over the 5-year period, including supplies stored for at least 18 years. Once the calculation model had been proposed, different storage limit conditions were obtained after validating the methodology comparing the predicted allowable storage periods and conditions with the real ones.

## 2. Methodology

**Figure 1:**
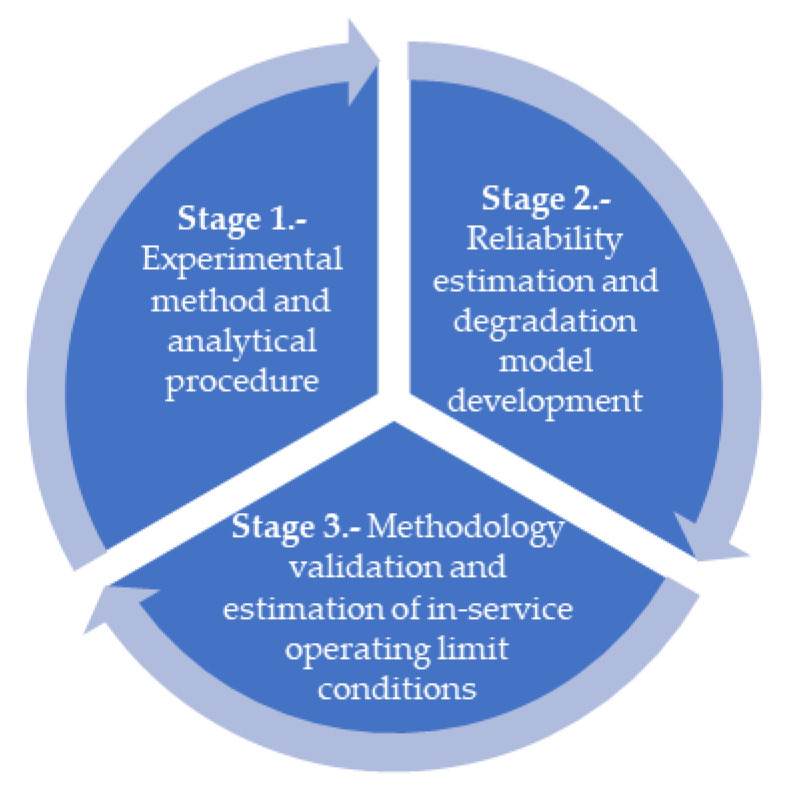
Methodology of analysis.

The methodology (Figure 1) is based on the analysis (Stage 1) of experimental data of Shore A hardness obtained during qualification processes (between 2014 and 2018) of recently manufactured (when they were measured) and previously stored NBR O-rings. Thus, by adapting the Arrhenius model for thermal aging, along with the activation energies indicated in the standard EPRI TR 1,009,748 [33], predictions (Stage 2) based on three scenarios are considered—very conservative, moderately conservative, and minimally conservative. Finally, a validation methodology is performed along with the estimation of in-service durability and the determination of critical storage conditions (Stage 3).

### 2.1. Stage 1: Experimental Method and Statistical Processing of Data

#### 2.1.1. Experimental Procedure

The experimental procedure consisted of a dimensional checking (a) and polymer composition characterization (b) before performing a Shore A hardness measurement (c), with all testing performed at 22 ± 1 °C and 25 ± 5% of humidity, using a thermo-hygrometer Testo 608-H1 (Testo SE & Co. KGaA, Lenzkirch, Germany). Table 1 shows the expected Shore A hardness of NBR O-rings and the hardness acceptance criterion along with the homogenous dimensions of O-rings.

(a) Dimensional Checking

**Figure 2:**
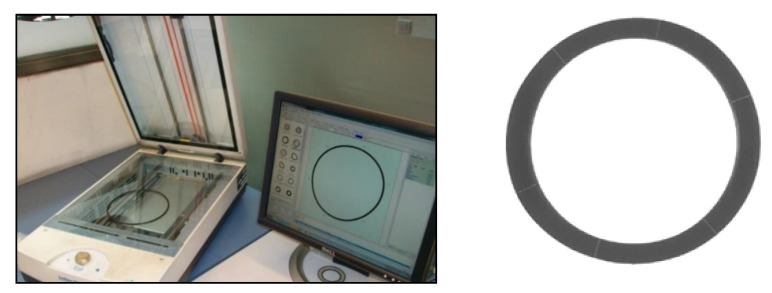
Dimensional checking procedure and example of measurement.

For reproducibility and comparison purposes, O-rings with identical nominal dimensions (reported in Table 1) were analyzed in the present study. The dimensional checking was performed using as an acceptance criterion of just ±1% for external and internal diameters, and therefore, for thickness. The thickness seems to be a critical aspect that could substantially influence the measurement as many studies have demonstrated [34]. In addition, the standardized procedure according to ASTM D2240 [35] and some authors [36] recommended that thickness should be at least equal to 6 mm. The measurements were performed on more than 140 O-rings from 14 different supplies and on an additional batch consisting of previously stored O-rings. The dimensional measurements were carried out using an equipment ScanMaker 9800XL PLUS TMA1600 III (Microtek, Hsinchu, Taiwan) as it is shown in Figure 2.

(b) Polymer Composition Characterization

**Figure 3:**
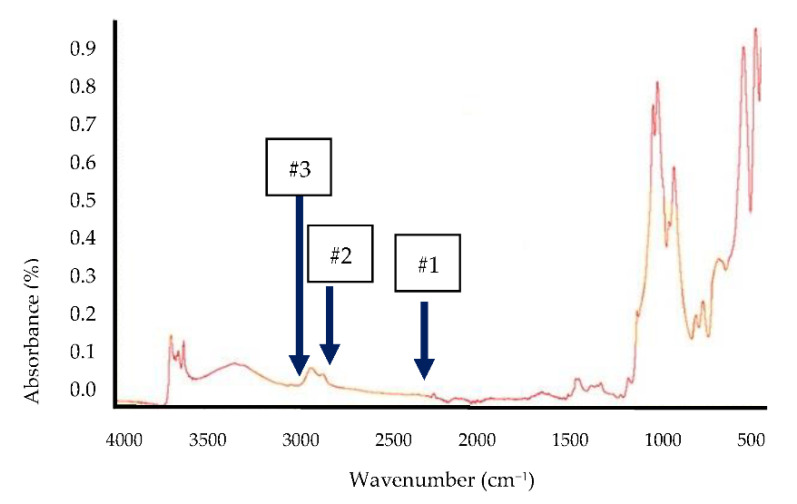
FTIR spectra.

Before performing the hardness test, each O-ring was also analyzed to assess the composition of components. In this case, the expected (and the acceptance) criterion was acrylonitrile butadiene rubber (NBR). The technique used was the Fourier-transform infrared spectroscopy (FTIR) that is based on the concept of absorption of infrared radiation by sample. The resulting signal at the detector is a spectrum that characterizes the polymer analyzed and, therefore, it allows composition data to be obtained [37]; this technique is the method used to determine if the O-ring composition is the expected one and, therefore, if the component is ready to continue the characterization process (hardness test, in this case study). The equipment used was a Nicolet 5700 (Thermo Electron Corporation, Waltham, MA, USA). Thus, spectra of the NBR components were recorded over a wavenumber range of 4000–500 cm^−1^, with 32 scanning times at a resolution of 4 cm^−1^. Figure 3 exhibits the FTIR spectra along with the indicated characteristic peaks of NBR provided in Table 2.

Once the composition was checked, the Shore A hardness according to ISO 868 [31] was performed.

(c) Hardness Test

The shore hardness was measured by the depth of indentation caused by a rigid ball under a spring load or dead load, the indentation being converted to hardness degrees on a scale ranging from 0 to 100. The reading from a dead-load hardness meter is called the international rubber hardness degree (IRHD). The spring-loaded meter gives Shore A values [39]. The energy absorbed by the sample material on impact is then related to the product of a “dynamic yield pressure” and the volume of the indent [40]. As indicated by Brown [41], the test results are affected by the operator, the time of application, and the deviations from perfectly elastic despite correct calibration and measurements according to the standard testing procedure. Spetz [42] examined the repeatability of hardness measurements on rubber materials and concluded that the operator was the main source of variability [43]. Thus, during the indentation experiments, hardness changes not only with the hold time but also with loading and unloading rate [44].

**Figure 4:**
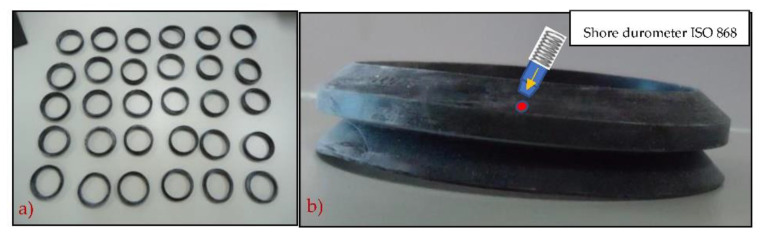
(**a**) Detailed of a batch of tested O-rings and (**b**) side view of O-ring and position of the Shore hardness indenter.

Figure 4 provides a detail of the O-rings (a) along with the position for the indentation and (b) the testing measurement locations (TML). All O-rings measured exhibited the same geometrical (nominal) characteristics (external and internal diameter and thickness).

**Figure 5:**
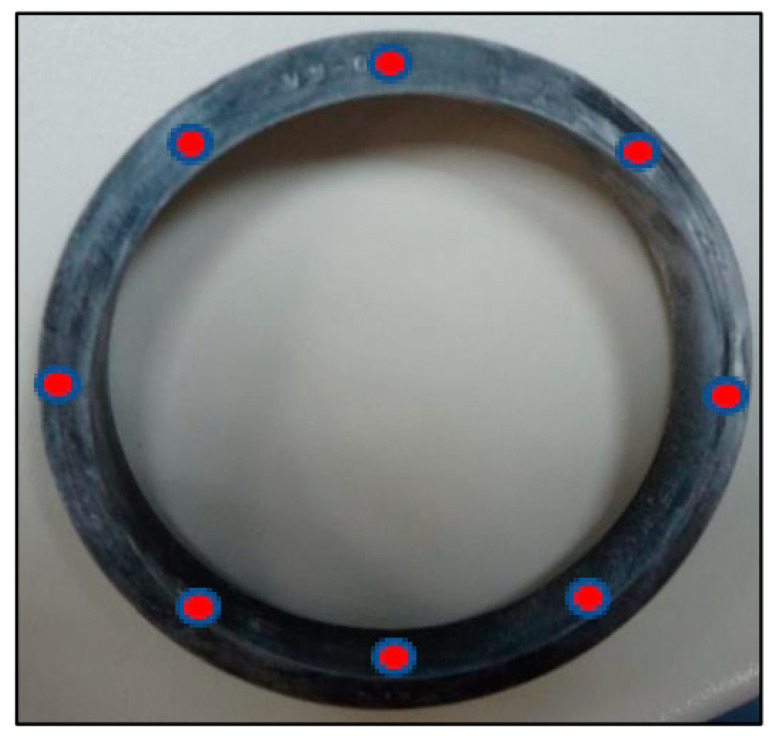
Front view of O-ring and position of indentations (TML).

Therefore, all measurements were performed by the same operator, using calibrated equipment and not repeating the hardness measurement at the same place because it provides permanent local changes in the material [45,46]. Figure 5 shows the testing measurement locations (TMLs) used in each characterized O-ring. The hardness testing was performed by using a durometer Zwick Roel Digi-Test Shore A/B/O (Zwick Roel, Ulm, Germany).

Once all hardness data was collected, an analytical procedure was carried out.

#### 2.1.2. Analytical Procedure

Hardness dispersion of rubber samples can be statistically well described by a normal distribution model [47]. Thus, Shore A hardness was fitted by a random normal distribution. Certainly, the Gaussian or normal distribution is the most-established model to characterize quantitative variation of original data. Accordingly, data are typically summarized using the arithmetic mean and the standard deviation, by $\bar{\mu }\pm \sigma$ [48]. Additionally, this type of representation allowed us to easily compare the mean and deviation among different supplies (from 2014 to 2018). The expression [49] for the one-dimensional normal density is often written according to Equation (1).

(1) $$f\left(HSA\right)=\frac{1}{\sqrt{2\pi \sigma }}\cdot e^{-\frac{{\left(HSA-\mu \right)}^{2}}{2\sigma^{2}}}$$

where *HSA* is Shore A hardness, *μ* is the mean, and *σ* is the standard deviation

**Figure 6:**
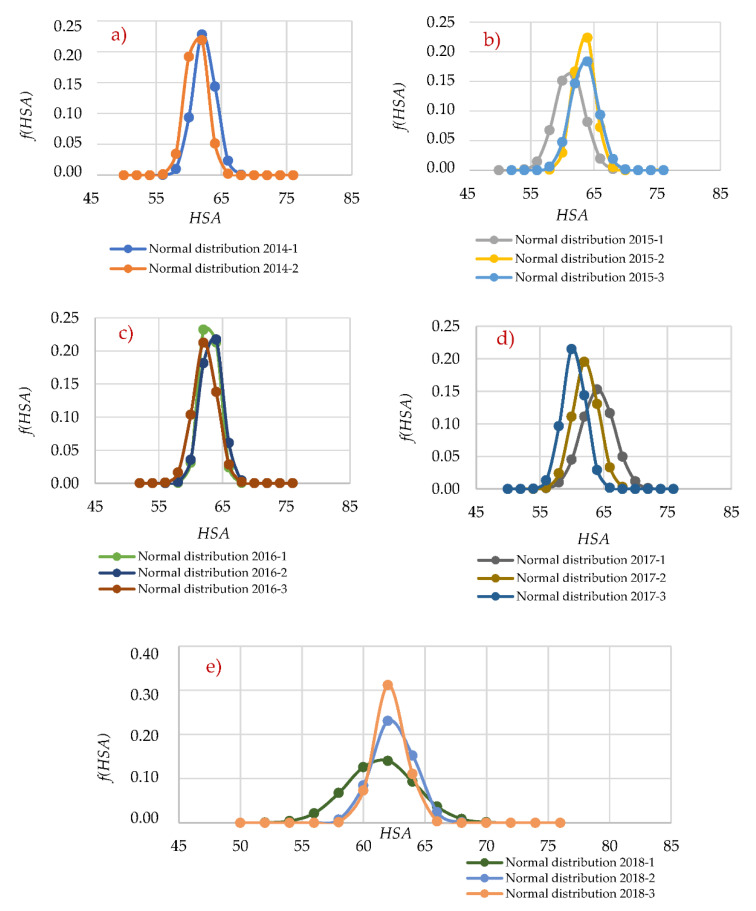
Normal distribution of hardness Shore A for recently manufactured O-rings. (**a**) 2014 supplies, (**b**) 2015 supplies, (**c**) 2016 supplies, (**d**) 2017 supplies, and (**e**) 2018 supplies.

Figure 6 provides the normal distribution (density function versus measured hardness) for each supply.

Table 3 shows mean values (*μ*) along with the standard deviation (*σ*) between measurements in each group of supplies (batches) and percentage variation in hardness of these measurements of the O-rings (as supplied) compared to stored O-rings.

The hardening experienced by the O-rings was between 11.80 and 15.98% with a difference in means (recently manufactured versus stored ones) of 13.81% (according to Table 4). Consequently, Table 4 shows the mean value of more than 140 Shore A hardness measurements made during the period between 2014 and 2018. Likewise, the study incorporated 12 hardness tests on stored O-rings without a defined date [50]. Nevertheless, it is known that they were entered into inventory in 2000 and that they could be dated form as early as 1994 (calculated on the test date in 2018).

**Figure 7:**
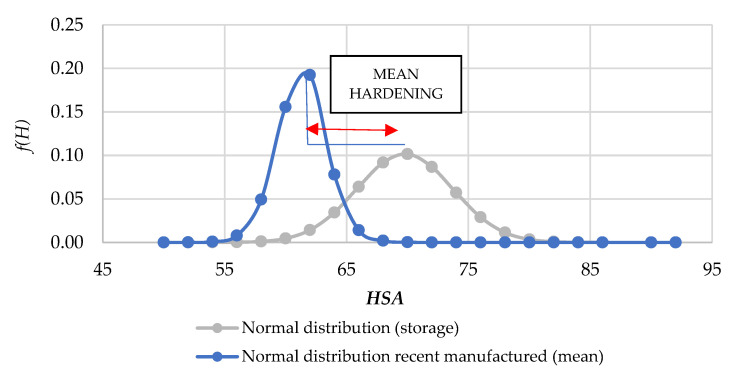
Hardening: difference between recently manufactured and storage normal distributions.

A recent study stated that the mean hardening of some NBR samples after 18 years was of 11.66% [51]; therefore, there is coherence in the observed results, especially considering that the analyzed storage time was between 18 and 24 years. Consequently, this could be considered as a validated starting point to perform further methodological analysis. Using a normal representation, Figure 7 provides the mean hardening for recently manufactured and stored O-rings.

**Figure 8:**
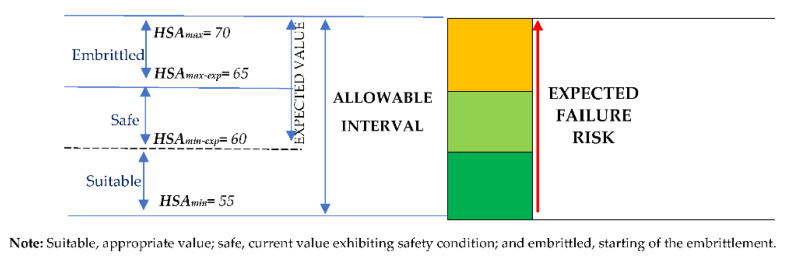
Hardness intervals and their correspondence to the risk of failure (loss of integrity due to aging).

Figure 7 shows that the mean value for recently manufactured (*HSA_mean_* = 61.33) O-rings was close to the expected value of 60 Shore A, whereas the mean value for stored (*HSA_mean_* = 69.78) O-rings was very close to the maximum allowable hardness (*HSA_max_* = 70 Shore A). Experimental findings demonstrated that O-rings with a Shore A hardness near to 70 are prone to failure [52]; subsequently, a three differentiated ranges are defined (Figure 8) as suitable, safe, and embrittled zones according to O-ring hardness. Thus, the risk associated with O-ring failure increases with Shore A hardness.

**Figure 9:**
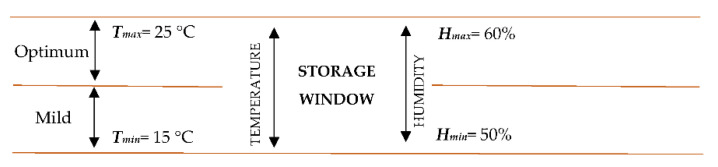
Storage window for temperature and humidity.

There are a lot of characteristics that have to be considered when a polymer candidate is evaluated for an application in the harsh environment of a nuclear plant. Some of these features are related mainly to thermal and radiation tolerance and its influence on mechanical properties [53]. Thus, defined normal conditions (Figure 9) allow us to consider different scenarios depending on the parameters’ variability inside the constructed range.

After defining the storage parameters’ window and the ranges of hardness associated to degradation and risk of failure (ranges that do not fulfill the safety function; i.e., preventing leakages), an Arrhenius-based model was developed [50], according to Equation (2), to correlate operation (or storage) time with operation (or storage) temperature:

(2) $$t_{s}=t_{a}\cdot exp\left[\frac{E_{a}}{k}\left(\left(\frac{1}{T_{a}}\right)-\left(\frac{1}{T_{s}}\right)\right)\right]$$

where *t_s_* is the estimated lifetime in service (hours), *t_a_* is the time considering acceleration in aging/degradation (hours), *T_s_* is the normal operating temperature (K), *T_a_* is the hardening temperature (K), *E_a_* is the activation energy (eV), and *K* is the Boltzmann constant (0.8617·10^4^ eV/K).

The activation energy used in the calculation was provided by EPRI TR 1,009,748 [26], that for NBR is equal to 0.88 eV.

As was mentioned before, 14 new supplies were compared with a large, stored batch. There is, therefore, an uncertainty related to the manufacturing date of stored O-rings. Considering this uncertainty about the date of manufacture of the previously stored O-rings, three scenarios have been defined for the analysis—very conservative, moderately conservative, and minimally conservative. Subsequently, for the conservative interval, it was considered that the age of O-rings was 24 years, for the middle one (moderately conservative), 22.5 years and for the least conservative one, 18 years old (calculated on the test date in 2018).

## 3. Results and Discussion

Once the testing and the first statistical analysis (Stage 1) had been performed, a reliability estimation was carried out in order to develop a degradation model with respect to storage conditions, such as temperature or time (Stage 2).

### 3.1. Stage 2.: Reliability Estimation and Degradation Model Development

Considering a well-stablished correlation between hardening and temperature, the Arrhenius model can be rearranged [52], according to Equation (3), to obtain in-service durability (*t_s_*):

(3) $$t_{s}=t_{a}\cdot exp\left[\frac{E_{a}}{k}\left(\left(\frac{1}{HSA_{augm}}\right)-\left(\frac{1}{HSA_{exp}}\right)\right)\right]$$

where *HSA_augm_* is the Shore A hardness augmented due to thermal aging with respect to *HSA_exp_* (expected *HSA*).

**Figure 10:**
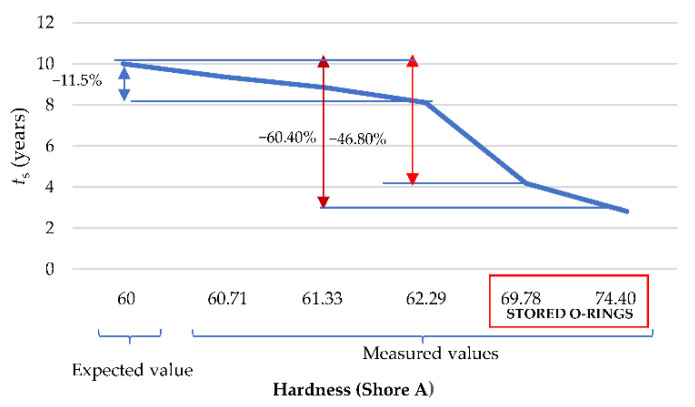
In-service durability as a function of the hardness.

Thus, with the measured hardness for recently manufactured and stored O-rings, in-service durability was calculated (Figure 10).

The storage conditions (and their associated hardening) have a direct effect on in-service durability, reducing it by up to 60.40%. Thus, time to integrity loss (*TTIL*), considered as the time in operation where a Shore A hardness equals 65 (beginning of the embrittlement; according to Figure 9), can be calculated by using the Equation (4).

(4) $$TTIL=lim_{HSA_{augm}\to 65\;\;\;\;\;\;\;\;\;}t_{a}\cdot exp\left[\frac{E_{a}}{k}\left(\left(\frac{1}{HSA_{augm}}\right)-\left(\frac{1}{HSA_{exp}}\right)\right)\right]$$

It can be concluded that the materials’ response could be considered similar to a previous operation time of 6 years (52,560 h). If we considered the extreme case in which 70 shore A is reached, *TTIL* would be equal to 4 years (35,040 h). On the other hand, if a new reformulation of Arrhenius model is performed, Equation (5) provides the hardening as a function of the durability of recently manufactured O-rings (*t_s_*) and stored ones (*t_a_*) and the measured hardness once stored (HSA).

(5) $$Hardening\;\left(\%\right)=\frac{100\cdot k\cdot HSA}{E_{a}}\cdot ln\frac{t_{S}}{t_{a}}$$

**Figure 11:**
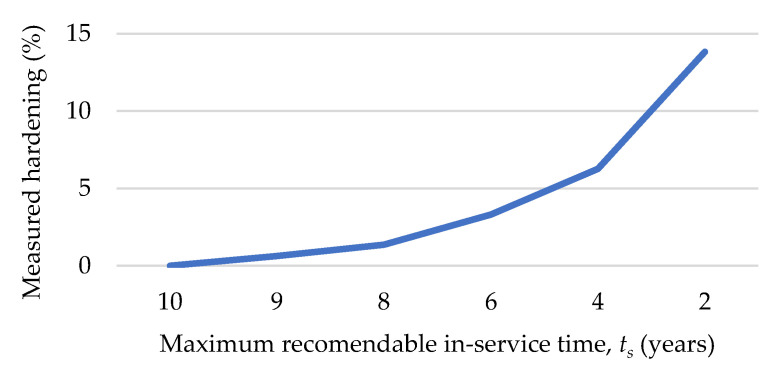
Maximum recommendable in-service time (*t_s_*) as a function of hardening (from hardness values).

Subsequently, Figure 11 exhibits the maximum recommendable in-service time as a function of hardening (from hardness values). This representation was performed according to Equation (5).

As Figure 11 indicates, the measured hardening can be a useful parameter to estimate the maximum recommendable in-service time. Considering that no measurable hardening (i.e., a value of 60 *HSA*) implies the maximum in-service time (10 years, that it is the usual qualified lifetime for O-rings in the nuclear industry), a hardening of 5% generates a reduction of the recommended in-service time of 50% (i.e., a recommended time of use of 5 years), while a hardening of 10% implies a usability for only 3 years.

Using an exponential distribution for the degradation (according to the Arrhenius model), the reliability function *R*(*t*) can be calculated [24] according to Equation (6).

(6) $$R\left(t\right)=e^{-\lambda t}$$

where λ is the failure rate, calculated as $\lambda =\frac{1}{TTIL}$ and *t*, the considered time.

**Figure 12:**
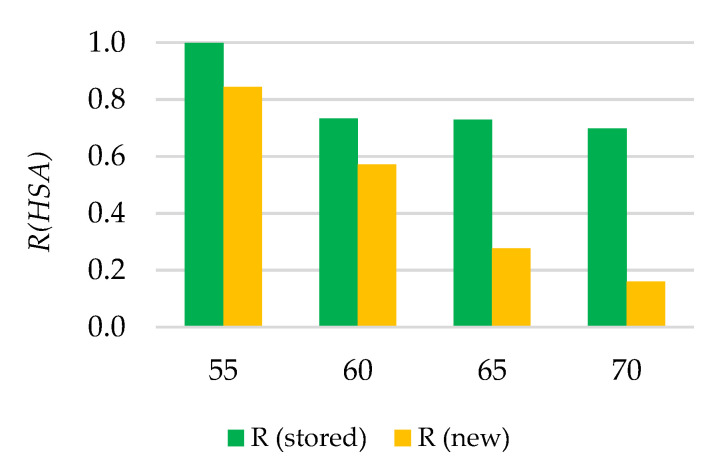
Reliability as a function of measured hardness for new (recently manufactured when measured) and stored O-rings.

The reliability of new (recently manufactured) and stored O-rings, represented as a function of the hardness, is shown in Figure 12.

**Figure 13:**
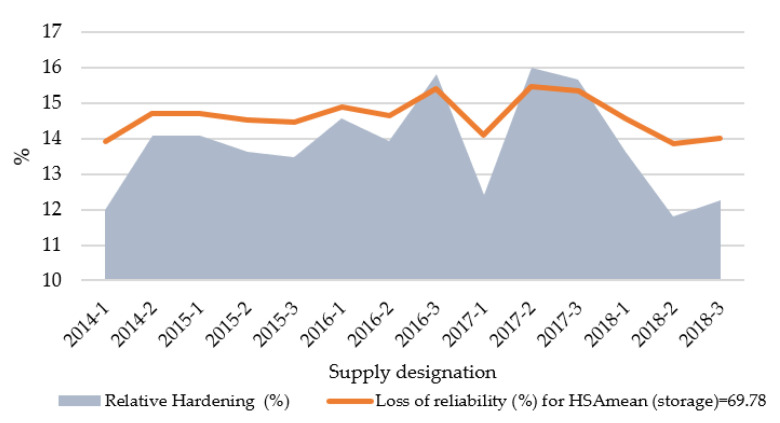
Relative hardening and loss of reliability (stored versus recently manufactured material).

Figure 13 provides the relative hardening of stored O-rings with respect to each supply of recently manufactured O-rings (shown in x axis). A loss of reliability for the upper limit of *HSA* established in 69.78 (mean value of hardness for stored O-rings) is simultaneously represented to be compared with the relative hardening for each recently manufactured supply.

As Figure 13 shows, the *R*(*t*) of stored manufactured O-rings was greater than the *R*(*t*) of the recent ones, independently of the hardness range. Nevertheless, the loss of reliability for recently manufactured O-rings was bigger when the hardening was greater. This is very reasonable because a hardening found in a recently manufactured O-rings probably implies a defective mechanical integrity or a degraded composition, while the same value for a stored O-ring just indicates that a hardening process took place. On the other hand, probability of failure distribution (*POF* (*t*)) can be calculated [24] according to Equation (7).

(7) $$POF\;\left(t\right)=1-e^{-\lambda t}=1-R\left(t\right)$$

**Figure 14:**
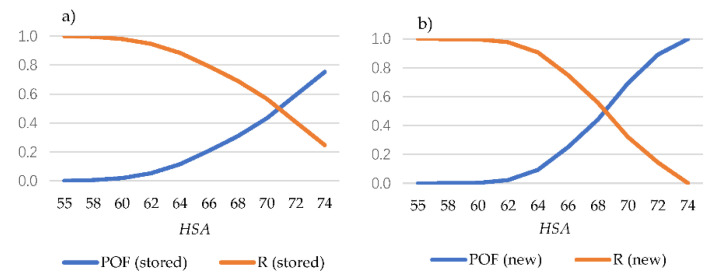
Reliability and probability of failure as a function of the measured hardness for the (**a**) stored and (**b**) recently manufactured O-rings.

Thus, *R*(*t*) and *POF* (*t*) related to the performance fulfillment are represented (Figure 14) as a function of the measured hardness.

**Figure 15:**
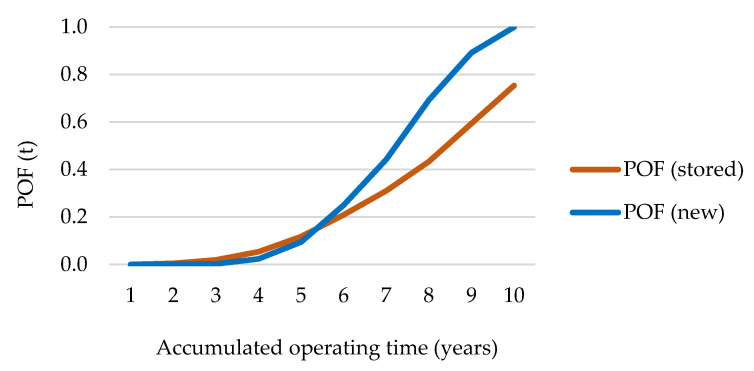
Probability of failure according to the accumulated operating time.

As a degradation and, therefore, a loss of integrity is expected when hardening takes place, in the case of stored O-rings (Figure 14a), a loss of more than the 20% of reliability is presented from a value equal to 65 Shore A hardness, being more than 50% from 70 shore A hardness. On the other hand, in the case of recently manufactured O-rings a hardness equal to 60 ± 5 is expected (as typically required by manufacturer; see Figure 9) showing a good reliability. Thus, a loss of 20% of reliability takes places when hardness is increased up to 65 shore A hardness, and from 68 HSA when the reliability is less than 50% (Figure 14b). In addition, POF as a function of the operation time (years), for both recently manufactured and stored O-rings (with a hardness close to 65 HSA) is shown in Figure 15.

**Figure 16:**
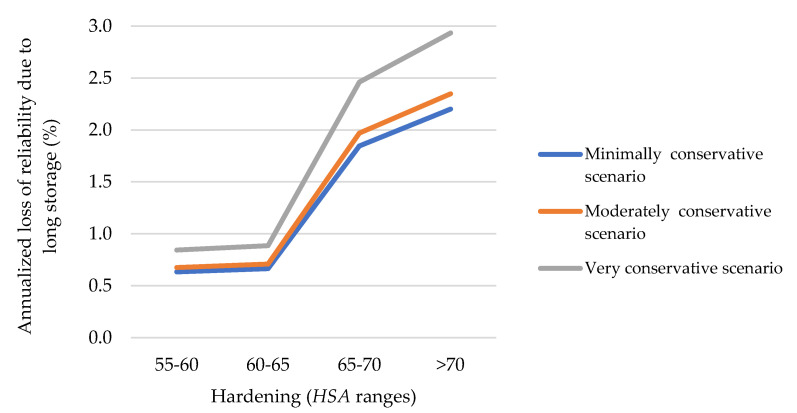
Annualized loss of reliability due to a long storage.

*POF* (*t*) represented in Figure 15 indicates how the accumulated in-service time affects the risk of failure (losing reliability). During the three first years of operation the increase of *POF* is practically insignificant. Nevertheless, from this point, and especially, from 5 years of operation, the *POF* increased from 10% to 20% at approximately 6 years (for new and stored). From 6 years, the behavior of curves (for new and stored) are more different. In the case of stored ones, there is a linear progression up to reaching a *POF* equal to 0.78 at 10 years; while, in the case of the new ones, the *POF* is practically 100% when they reach an accumulated in-service time equivalent to 10 years. Seen from another point of view, the annualized loss of reliability can be quantified as a function of the hardening (or the measured value of hardness). As the last compared supply was dated in 2018, three comparative scenarios were established (very conservative, 24 years; medium, 22.5 years; and minimally conservative, 18). Thus, Figure 16 provides the annualized loss of reliability considering the three scenarios of analysis; since the loss of reliability is considered, in this case, to be due to the storage (and, therefore, the comparison of the three scenarios needs to be more precise).

In the range of 55–65 *HSA*, the loss of reliability due to storage was small, with hardly any difference between 55 and 60 and 60 and 65 (zone of acceptable values). The loss of reliability accelerated in the range 65–70 (the slope is greater), being higher from 70 Shore A. Equation (8) provides the time to damage (*TTD*) that is calculated from *TTIL* obtained from Equation (4).

(8) $$TTD=-\frac{ln\left(R\right)}{\lambda }\;\;\;\forall \;0<R\leq 0.99$$

On the other hand, using a defined safety factor (*SF*), a recommended replacement interval (*RPI*) can be calculated using the Equation (9).

(9) RPI=SF·TTD

According to Equations (8) and (9) and as function of different values of maximum allowable loss of reliability, *TTD* and *RPI* are obtained for recently manufactured O-rings (as the worst favorable scenario) with HSA > 65. This is presented in Table 5.

Thus, applying this model, if O-rings are replaced in annual operation of maintenance, the reliability of O-rings with a hardness of 65 shore A is 0.85, whereas in the case of O-rings with a hardness of 68 shore A it is 0.78. Nevertheless, the recommendation is to use O-rings with a hardness lower than 60 HSA, to ensure a reliability above 0.90.

### 3.2. Stage 3.: Methodology Validation and Estimation of In-Service Operating Limit Conditions

Table 6 shows the maximum temperature obtained using Equation (1) and the calculation parameters indicated in Note *^3^ (at the bottom of the Table) and considering the three scenarios (as defined in Section 3.1)

In view of the results presented in Table 6, it can be concluded that the limit conditions for prolonged storage considering any of the three contemplated scenarios would be above the real conditions. That is, even in the case of the least conservative scenario, the maximum temperature predicted by the model is 25.17 °C, which is slightly higher than the maximum real temperature (according to Note * ^1^ in Table 4 = 20 ± 5 °C).

On the other hand, a validation (Table 7) is performed to check if the analyzed assumptions stated in the analytical procedure (18, 22.5, and 24 years) and the maximum allowable hardness value according to the catalog would be reached for these NBR gaskets, that is, a value of 70 Shore A [42].

**Figure 17:**
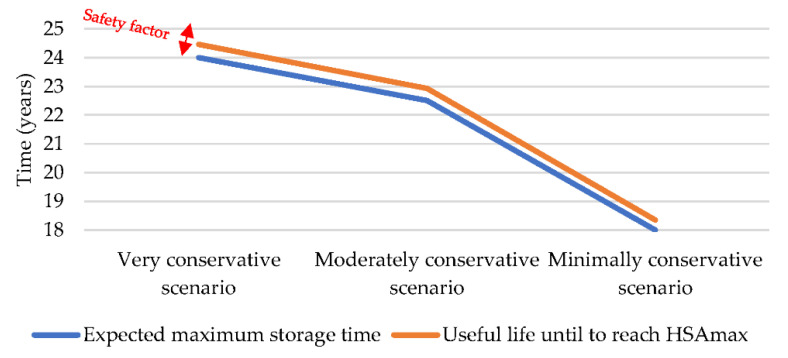
Validation and demonstration that the model provides a safety factor.

Adapting the model to predict in each of the three scenarios which maximum allowable hardness value (70 Shore A) (defined as the upper limit) would be reached, it was verified that for any of the scenarios the upper limit value is above the considered storage time premise (18.35 > 18 years for the least conservative scenario, 22.93 > 22.50 years for the medium scenario, and 24.46 > 24 years for the most conservative scenario). Therefore, it is possible to validate the model by ensuring that in the predictions (both for temperature ranges and for storage times) the allowable limit value of 70 Shore A is not reached in any case. Finally, an analysis to validate the methodology was performed (Figure 17) on the basis of the representation of the expected maximum storage time (using the three considered scenarios) versus the time to reach the *HSA_max_* (70 HSA). In addition, the starting data (hardness values) showed coherence with another experimental work such as the one of Zhong et al., that provides similar embrittlement by storage for a period of 18 years [51].

## 4. Conclusions and Future Work

The major conclusions resulting from this work can summarized as follows:

- The measured hardening can be a useful parameter to estimate the maximum recommended in-service time. A hardening of 5% generates a reduction of the recommended in-service time of 50% (i.e., a recommended time of use of 5 years), while a hardening of 10% implies a usability of only 3 years.
- The storage (and its associated hardening) of the NBR O-rings has a direct effect on the in-service durability, reducing this by up to 60.40%. Thus, the calculated time to integrity loss (*TTIL*), which is considered as the time in operation where a Shore A hardness equal to 65 is reached (beginning of the embrittlement), is 6 years (52,560 h).
- During the first three years of operation the increase of *POF* is practically insignificant. Nevertheless, from this point, and especially, from 5 years of operation, the *POF* increases from 10% to 20% at approximately 6 years (for new and stored).
- From 6 years of operation, the behavior of curves (for new and stored) are very different. In the case of stored ones, there is a linear progression up to reaching a *POF* equal to 0.78 at 10 years, while, in the case of the new ones, the *POF* is practically 100% when an accumulated in-service time equivalent to 10 years is reached.
- A validation of the methodology was performed by comparing the predicted allowable storage periods and conditions with the real ones. Thus, applying this model, if O-rings are replaced in annual operation of maintenance, the reliability of O-rings with a hardness of 65 shore A is 0.85, whereas in the case of O-rings with a hardness of 68 shore A it is 0.78.
- From the study, the general recommendation is using O-rings with a HSA less than 60 HSA, to ensure a reliability above 0.90. Finally, it was proved that the storage strategies of our nuclear power plants are successful, perfectly meeting the expectations of suitability and functionality of the components when they are installed after storage.

Finally, this methodology can be used in the future to analyze the suitability of other polymers after a long storage period.

## Figures and Tables

**Table 1:** Dimensional characteristics of O-rings.

Supply Description	Composition	Expected Shore A Hardness	Typical Hardness Acceptance Criteria	External Diameter, *Φext* (mm)	Internal Diameter, *Φint* (mm)	Thickness (*t*) (mm)
O-rings (type V)	NBR	60	60 ± 5	110	100	8

**Table 2:** Characteristic bonds of NBR as a function of wave number in the Fourier transform infrared spectroscopy test [38].

Polymer	Number of Peak in Figure 3	Wave Number (cm^−1^)	Indication/Type of Bond Identified
NBR	#1	2236	Stretching for –C=N
	#2	2851	–C–H stretch of –CH3
	#3	2922	–C–H stretch of –CH2

**Table 3:** Variation of every new supply hardness compared with stored ones and group standard deviation in measurements.

References (Year and Correlative Number)	Shore A—Mean Hardness	Percentage Variation in Hardness Compared to Stored O-rings	Standard Deviation
2014-1	62.32	−11.98	1.72
2014-2	61.17	−14.09	1.59
2015-1	61.19	−14.08	2.33
2015-2	61.42	−13.62	1.68
2015-3	61.50	−13.47	2.11
2016-1	60.92	−14.56	1.38
2016-2	61.25	−13.93	1.66
2016-3	60.25	−15.82	1.86
2017-1	62.08	−12.40	2.61
2017-2	60-17	−15.98	2.04
2017-3	60.33	−15.66	1.83
2018-1	61.42	−13.62	2.78
2018-2	62.42	−11.80	1.68
2018-3	62.17	−12.25	1.27
Stored batches	69.78	−	2.62

**Table 4:** Experimental data analyzed in this work ^1^.

Supply Description	Shore A Hardness (Mean Value)
New supplies (acquired between 2014 and 2018)	61.33
Supplies stored for at least 18 years	69.78
**Evaluation Parameter**	Hardening *^1^ (Difference between Means) (%)
New to storage supplies comparison	13.81

Note *^1^: Storage conditions: temperature = 20 ± 5 °C; relative humidity = 50–60% [42].

**Table 5:** Time to Damage (*TTD*) estimation and recommended replacement interval (*RPI*) for O-rings with *HSA* > 65.

Maximum Allowable Loss of Reliability	*TTD* (Years)	*RPI* (Years) *^2^
0.2	1.33	12
0.3	2.14	19
0.4	3.06	27

Note *^2^: a *SF* equal to 0.75 was used (but this value can be fit according to the acceptable risk defined by the plant’s owner).

**Table 6:** Prediction of the maximum allowable storage temperature according to the Arrhenius model.

Scenario of Analysis	Maximum Allowable Storage Temperature (°C) *^3^	Validation According to the Established Hypotheses
Very conservative	27.50	>upper limit of T = 20 ± 5 °C
Moderately conservative	26.31	>upper limit of T = 20 ± 5 °C
Minimally conservative	25.17	>upper limit of T = 20 ± 5 °C

Note *^3^: The following parameters have been used for the calculation: normal operating temperature (*T_s_*) = 33 °C; operation time= 10 years; activation energy (*Ea*) according to EPRI TR 1,009,748 for NBR= 0.88 [26]. Note *^3^ Values > controlled room temperature (T = 20 ± 5 °C) [42].

**Table 7:** Results of the application of the Arrhenius-based model and validation.

Analysis Scenario	Time (Years) to Reach the Maximum Allowable Hardness (70 Shore A)	Validation Criterion (Valid if it is “Above”)
Minimally conservative	18.35	18
Moderately conservative	22.93	22.5
Very conservative	24.46	24

## Data Availability

The data presented in this study are available on request from the corresponding author.

## References

[1] Cruz Sanchez F.A., Boudaoud H., Hoppe S., Camargo M. (2017). Polymer recycling in an open-source additive manufacturing context: Mechanical issues. Addit. Manuf..

[2] Vidakis N., Petousis M., Maniadi A., Koudoumas E., Vairis A., Kechagias J. (2020). Sustainable Additive Manufacturing: Mechanical Response of Acrylonitrile-Butadiene-Styrene over Multiple Recycling Processes. Sustainability.

[3] Paajanen A., Sipilä K. (2015). Modelling Tools for the Combined Effects of Thermal and Radiation Ageing in Polymeric Materials.

[4] Burnay S.G. (2001). An overview of polymer ageing studies in the nuclear power industry. Nucl. Instrum. Methods Phys. Res. B.

[5] Zaghdoudi M., Kömmling A., Jaunich M., Wolf D. (2019). Scission, Cross-Linking, and Physical Relaxation during Thermal Degradation of Elastomers. Polymers.

[6] Frigione M., Naddeo C., Acierno D. (2001). Cold-curing epoxy resins: Aging and environmental effects. I-Thermal properties. J. Polym. Eng..

[7] Bouaziz R., Truffault L., Borisov R., Ovalle C., Laiarinandrasana L., Miquelard-Garnier G., Fayolle B. (2020). Elastic Properties of Polychloroprene Rubbers in Tension and Compression during Ageing. Polymers.

[8] Mao L., Davies B., Jackson L. (2017). Application of the sensor selection approach in polymer electrolyte membrane fuel cell prognostics and health management. Energies.

[9] Sikorska J.Z., Hodkiewicz M., Ma L. (2011). Prognostic modelling options for remaining useful life estimation by industry. Mech. Syst. Signal Pract..

[10] Cubillo A., Perinpanayagam S., Esperon-Miguez M. (2016). A review of physics-based models in prognostics: Application to gears and bearings of rotating machinery. Adv. Mech. Eng..

[11] Bowler N., Liu S. (2015). Aging Mechanisms and Monitoring of Cable Polymers. Int. J. Progn. Health Manag..

[12] Khan I., Hussain G., Al-Ghamdi K.A., Umer R. (2019). Investigation of impact strength and hardness of UHMW polyethylene composites reinforced with nano-hydroxyapatite particles fabricated by friction stir processing. Polymers.

[13] EPRI CGI-OR02 (1992). Commercial Grade Item Evaluation for National O-Rings.

[14] Klingender R.C., Klingender R.C. (2008). Acrylonitrile Butadiene Rubber. Specialty Elastomers.

[15] Degrange J.-M., Thomine M., Kapsa P.H., Pelletier J.M., Chazeau L., Vigier G., Dudragne G., Guerbé L. (2005). Influence of viscoelasticity on the tribological behaviour of carbon black filled nitrile rubber (NBR) for lip seal application. Wear.

[16] Kapitonov E.A., Petrova N.N., Mukhin V.V., Nikiforov L.A., Gogolev V.D., Shim E.L., Okhlopkova A.A., Cho J.-H. (2021). Enhanced Physical and Mechanical Properties of Nitrile-Butadiene Rubber Composites with N-Cetylpyridinium Bromide-Carbon Black. Molecules.

[17] Bafna S. (2013). Factors influencing hardness and compression set measurements on O-rings. Polym. Plast. Technol. Eng..

[18] Rodríguez-Prieto A., Camacho A.M., Callejas M., Sebastián M.A. (2020). Fitness for service and reliability of materials for manufacturing components intended for Demanding Service Conditions in the Petrochemical Industry. IEEE Access.

[19] Frigione M., Lettieri M. (2020). Recent advances and trends of nanofilled/nanostructured epoxies. Materials.

[20] Rodríguez-Prieto A., Camacho A.M., Sebastián M.A., Yanguas-Gil A. (2019). Analysis of mechanical and thermal properties of elastomers for manufacturing of components in the nuclear industry. Procedia Manuf..

[21] Kömmling A., Jaunich M., Pourmand P., Wolff D., Hedenqvist M. (2019). Analysis of O-Ring seal failure under static conditions and determination of end-of-lifetime criterion. Polymers.

[22] White J.R. (2006). Polymer ageing: Physics, chemistry or engineering? Time to reflect. C. R. Chim..

[23] Moraczewski K., Stepczynska M., Malinowski R., Karasiewicz T., Jagodzinski B., Rytlewski P. (2019). The effect of accelerated aging on polylactide containing plant extracts. Polymers.

[24] Rodríguez-Prieto A., Primera E., Callejas M., Camacho A.M. (2020). Reliability-based evaluation of the suitability of polymers for additive manufacturing intended to extreme operating conditions. Polymers.

[25] Csányi G.M., Bal S., Tamus Z.A. (2020). Dielectric measurement based deducted quantities to track repetitive, short-term thermal aging of Polyvinyl Chloride (PVC) cable insulation. Polymers.

[26] Azura A., Thomas A., Coveney V. (2006). Effect of heat ageing on crosslinking scission and mechanical properties. elastomer and components. service life prediction–progress and challenges. Elastomer and Components: Service Life Prediction—Progress and Challenges.

[27] Zaghdoudi M., Kömmling A., Jaunich M., Wolf D. (2020). Erroneous or Arrhenius: A degradation rate-based model for EPDM during homogeneous ageing. Polymers.

[28] Moon B., Jun N., Park S., Seok C.-S., Hong U.I. (2019). A study on the modified Arrhenius equation using the oxygen permeation block model of crosslink structure. Polymers.

[29] IAEA-TECDOC-1551 (2007). Implementation Strategies and Tools for Condition Based on Maintenance at Nuclear Power Plants.

[30] Van de Voorde M.H., Restat C. (1972). Selection Guide to Organic Materials for Nuclear Engineering.

[31] ISO 868 (2003). Plastics and Ebonite—Determination of Indentation Hardness by Means of a Durometer (Shore Hardness).

[32] Gargol M., Klepka T., Klapiszewski L., Podkoscielna B. (2021). Synthesis and Thermo-Mechanical Study of Epoxy Resin-Based Composites with Waste Fibers of Hemp as an Eco-Friendly Filler. Polymers.

[33] EPRI TR 1009748 (2005). Guidance for Accident Function Assessment for RISC-3 Applications.

[34] Bassi A.C., Casa F., Mendichi R. (1987). Shore A hardness and thickness. Polym. Test..

[35] ASTM D-2240 (2015). Standard Test Method for Rubber Property—Durometer Hardness.

[36] Siddiqui A., Braden M., Patel M.P., Parker S. (2010). An experimental and theoretical study of the effect of sample thickness on the Shore hardness of elastomers. Dent. Mater..

[37] Rodríguez-Prieto A. (2019). Ingeniería inversa y caracterización avanzada de materiales para el establecimiento de requisitos de aceptación en procesos singulares de dedicación. Nuclear España.

[38] Samantarai S., Nag A., Singh N., Dash D., Basak A., Nando G.B., Das N.C. (2018). Chemical modification of nitrile rubber in the latex stage by functionalizing phosphorylated cardanol prepolymer: A bio-based plasticizer and a renewable resource. J. Elastomers Plast..

[39] Chandrasekaran V.C. (2007). Essential Rubber Formulary, Formulas for Practitioners.

[40] Briscoe B.J., Sinha S.K., Swallowe G.M. (1999). Hardness and Normal Indentation of Polymers. Mechanical Properties and Testing of Polymers.

[41] Brown R. (2006). Physical Testing of Rubber.

[42] Spetz G. (1993). Improving precision of rubber test methods: Part 1—Hardness. Polym. Test..

[43] Vieira T., Lundberg J., Eriksson O. (2020). Evaluation of uncertainty on Shore hardness measurements of tyre treads and implications to tyre/road noise measurements with the Close Proximity method. Measurement.

[44] Slouf M., Strachota B., Strachota A., Gajdosova V., Bertschova V., Nohava J. (2020). Macro-, micro- and nanomechanical characterization of crosslinked polymers with very broad range of mechanical properties. Polymers.

[45] Petik F. (1990). Metrology of hardness: Past development and present state of the art. Measurement.

[46] Ibáñez García A., Martínez García A., Ferrándiz Bou S. (2020). Study of the influence of the almond shell variety on the mechanical properties of starch-based polymer biocomposites. Polymers.

[47] Liu Q., Shi W., Chen Z., Li K., Liu H., Li S. (2019). Rubber accelerated ageing life prediction by Peck model considering initial hardness influence. Polym. Test..

[48] Limpert E., Stahel W.A. (2011). Problems with using the normal distribution—and ways to improve quality and efficiency of data analysis. PLoS ONE.

[49] Stahl S. (2006). The evolution of the normal distribution. Math. Mag..

[50] Rodríguez-Prieto A., Callejas M., Primera E., Camacho A.M. (2020). Reliability and Thermal Aging of Polymers Intended to Severe Operating Conditions. Proceedings.

[51] Zhong R., Zhang Z., Zhao H., He X., Wang X., Zhang R. (2018). Improving thermo-oxidative stability of nitrile rubber composites by functional graphene oxide. Materials.

[52] Rodríguez-Prieto A. Evaluación analítica y Experimental de la Degradación por Almacenamiento de Juntas Elastoméricas Fabricadas como grado Comercial Destinadas en Aplicaciones Relacionadas con la Seguridad. Proceedings of the Virtual Meeting of the Spanish Nuclear Society.

[53] Rodríguez-Prieto A., Camacho A.M., Aragón A.M., Sebastián M.A., Yanguas-Gil A. (2018). Polymers selection for harsh environments to be processed using additive manufacturing techniques. IEEE Access.

